# Fang Filling

**Published:** 1859-09

**Authors:** W. H. Shadoan


### FANG FILLING.

BY W. H. SHADOAN.

MESSRS. EDITORS:—In the July number of the Register, I notice an article entitled "Fang filling," by Geo. Watt, which article is the first that has come under my notice that has so completely described my own process of filling teeth where the destruction of the nerve was absolutely necessary. The author of the article above referred to says, and very correctly too, that to fill all the pulp cavities of the teeth would be rather difficult. I find it so, and more particularly the anterior cavities of the molar teeth, when the posterior proximal surface is decayed, forming the only ingress to the pulp cavity.

I have been at a loss to know exactly how the advocates of fang filling manage to prepare the cavities of the molar teeth to the apex of the fangs, and introduce and properly manipulate the filling of the same. It does seem to me, when they say that they fill the entire canal of all the teeth, that they just mean to say, that they fill as far as they conveniently can, and that they do not intend to say that they fill the entire canal, at least this seems to me the most probable.

I would ask the question, *how do the advocates of fang filling manage in case they find a tooth to be filled*—say the second or third inferior molar—*that the posterior proximal surface is decayed and nerve is exposed, the destruction of which is inevitable*, and of course "the fangs will have to be filled, or the tooth cannot be saved." How do they excavate, cleanse, and properly manipulate a gold filling to the apex of the anterior fang of said tooth?

My practice has always been, since I have been in the practice, to fill all the fangs that I could where they needed it, but in no case have I been able to fill one of the above described cavities. Now the only reason that unless there is an opening made to the fang from the surface next it, the fang cannot be prepared, and consequently cannot be filled: beside, the nerve cavity is so small, that it takes a very nice operation to prepare the cavity to its apex. My experience has proved to me, that to fill the cavity of decay properly, and have all the nerve removed, and the nerve cavity properly cleansed, is about all that is necessary. One would ask how do you do this, and what is your success in your operations? My process is this, I destroy the nerve most generally with cobalt and creosote, sometimes however, I use other agents, such as arsenic, nitric acid, and chloride of zinc, but find that the cobalt and creosote are the best. After the nerve is destroyed, I remove it so far as I can, then wash out the cavity with Dr. Tefft's anæsthetic preparation, then with creosote and laudanum, mixed half and half, and lastly with creosote alone.

After it is well washed out, I wet a pledget of cotton and let it remain in the cavity about from six to ten or sometimes fifteen hours. I then find in nine cases out of ten, that the soreness is all dissipated, and the decayed matter sufficiently infused with the creosote to form an insoluble compound, which makes no real difference, whether it is removed or not. I find it necessary sometimes to treat the fangs of teeth for several days, but generally I am successful with the first operation.

I find that the safest plan is to treat as above until all the soreness is gone, then for fear of failure, only fill the pulp cavity to fangs, let it remain for a few days, and see whether it is going to cause any trouble, if not, then you may finish the operation with safety.

I have noted since the first of March, 1858, about thirty or forty cases, some of which were almost hopeless, as there had been alveolar abscesses formed at the roots of the teeth, prior to their application for an operation. Of the number noted, I do not think I have lost more than three or four, and I believe the cause of the loss of them was for want of time and care, not on account of the manner of procedure, or in the kind of treatment.

To say the least of it, I believe that I was as much to blame as any body or any thing; in other cases more aggravated, I have been successful, proving to me beyond a doubt, that if I would take the time, and my patients would all be punctual in their attendance, and would follow the directions, that I would not lose one case in twenty.

I would report several cases, but I hardly deem it necessary, as Dr. Watt, in his essay, gave several very interesting cases, that would correspond in the main with those I would report, so I will only report one or two at present, believing that it will be sufficient to convince the skeptical that there are other modes of doing business than that first conceived, or at least if our fathers used to carry a stone in one end of the sack to balance the other, it is no reason that we should do the same.

CASE I.—Mrs. M. H. S., aged 45. Health good, teeth bad, first bicuspid badly decayed. Destroyed nerve on the 7th of July, 1858, filled on the 9th.

Formula.—Destroyed the nerve with cobalt and creosote, extirpated as far as possible, treated with creosote on cotton, for two days, then plugged. September 8th, patient called suffering from the tooth just named. The conclusion I came to was, that there was some of the pulp remaining which had caused this trouble. I drilled a hole through the plug and into the nerve cavity, which emitted a very offensive matter, syringed the cavity with creosote and chloroform, separate; patient got well, and no more complaint until the 29th April, 1859, the filling came out. I put in another, since which time there has been no more complaint.

Case II.—R. Y., aged 32, teeth good, health good, called to have the left superior first molar extracted, after trying the second time to no purpose, he protested against any farther trial; I then destroyed the nerve with creosote and cobalt, and on the next day, January 20th, 1859, I filled the tooth. I have heard from him several times since, and at present there is no complaint.

